# Myopericytoma in an Infant—Imaging Characteristics of a Rare but Benign Entity

**DOI:** 10.5334/jbsr.3441

**Published:** 2024-01-11

**Authors:** Annika Herrtwich, Raf Sciot, Caroline Ernst

**Affiliations:** 1 Department of Radiology, UZ Brussels, VUB, Brussels, Belgium; 2 Department of Pathology, University Hospitals Leuven, KU Leuven, Leuven, Belgium; 3 Department of Pediatric Radiology, UZ Brussels, VUB, Brussels, Belgium

**Keywords:** Myopericytoma, soft tissue tumor, perivascular tumor, child

## Abstract

**Teaching point**: Myopericytoma is a rare soft tissue tumor but should be considered in the differential diagnosis of infants with a fast-growing perivascular tumor.

## Case History

A 3-month-old girl presents with swelling dorsomedially on the right foot. The swelling has been present since birth but displays exponential growth with blue discoloration. On physical examination, the lesion is exophytic, immobile, and painless.

**Figure 1:**
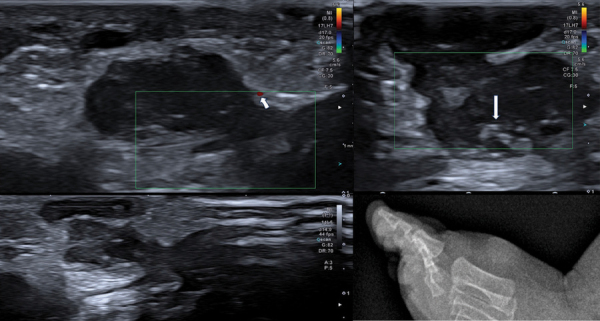
US images of the lesion showing little surrounding vascularization (arrow top left) and small calcifications (arrow top right). X-ray of the lesion (bottom right).

Ultrasound (Figure 1) shows a subcutaneous bilobar hypoechogenic, heterogeneous lesion with small calcifications. Only very little surrounding vascularization is visualized.

X-ray (Figure 1) of the foot presents a soft tissue swelling with small hyperdensities but no bony lesion.

**Figure 2:**
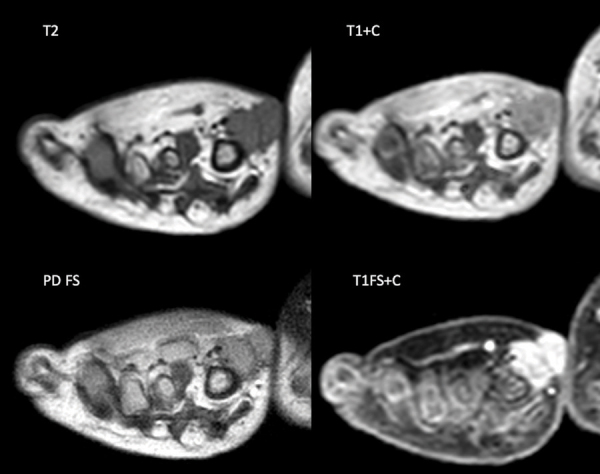
Coronal MRI images of the lesion showing isointense aspect on T2 (top left) and PD FS (down left) and bright enhancement on T1 (right).

Magnetic resonance imaging (MRI) (Figure 2) displays a bilobar subcutaneous lesion with a hypointense signal on T1, an isointense signal on PD, and T2 weighted images. The lesion shows an excessive, heterogeneous contrast enhancement. Diameters are approximately 11 × 9 × 11 mm.

**Figure 3:**
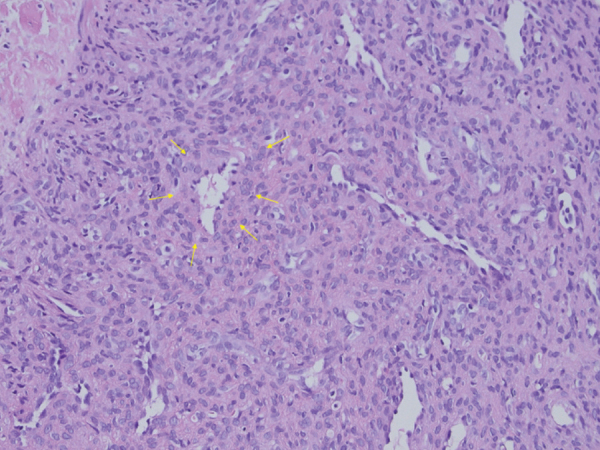
H&E stain of the lesion showing cuffs of pericytic cells around vessels (arrows). The pericytic cells express alfa-smooth muscle actin.

Biopsy (Figure 3) revealed small, branching capillaries surrounded by numerous spindle-shaped cells, suggestive of a benign perivascular myoid tumor like a glomus tumor or myopericytoma. Further tissue analysis resulted in the diagnosis of myopericytoma, confirmed by the presence of the pathological variant found in the PDGFRB gene.

The lesion is followed-up using ultrasound.

## Comments

Myopericytomas are rare benign perivascular soft tissue tumors, usually located subcutaneously on the lower limbs; however, they can appear anywhere on the body [1]. They can present at any age but are more common in younger patients.

Clinically, myopericytomas present as well-defined, solitary, palpable soft tissue masses that are usually painless and slow-growing. They might be present for years and generally have a good prognosis.

Very rarely, malignant myopericytomas have been reported, which are deeper and more invasive. They show nuclear pleomorphic and mitotic activity on tissue analysis [1].

Myopericytomas originate from smooth muscle-like, spindled myoid cells with concentric and perivascular growth patterns. They have similarities with other perivascular tumors described in the 2013 WHO Tumors of Soft Tissue and Bone classification. Previous WHO classifications did not categorize myopericytomas as a separate entity but within the hemangiopericytomas, leading to very limited literature for an already rare entity.

Radiographies of these lesions can show a dense soft tissue structure with possible small calcifications. On ultrasound, hypoechogenic soft tissue lesions with occasional calcifications can be seen. Computed tomography (CT) imaging can also reveal dystrophic mineralization and calcification. On MRI, lesions appear highly vascularized with vivid contrast enhancement. This can be homogenous or more heterogeneous with internal hemorrhages and sometimes dystrophic calcifications. Peritumoral edema can be variable.

Differential considerations include myofibroma, angioleiomyoma, infantile hemangiopericytoma, glomus tumor, or synovial sarcoma [1].
